# The Variability of Berry Parameters Could Be an Indicator of the Potential Quality of the Vineyard

**DOI:** 10.3390/plants13182617

**Published:** 2024-09-19

**Authors:** Zlavek Travanic-Fuentes, Gastón Gutiérrez-Gamboa, Yerko Moreno-Simunovic

**Affiliations:** 1 Centro Tecnológico de la Vid y el Vino, Facultad de Ciencias Agrarias, Universidad de Talca, 2 Norte 685, Casilla 747, Talca 3460000, Chile; zlavektf@gmail.com; 2 INIA Carillanca, Instituto de Investigaciones Agropecuarias, km 10 Camino Cajón-Vilcún s/n, Temuco Casilla Postal 929, Temuco 4880000, Chile

**Keywords:** berry composition, Cabernet Sauvignon, grape, harvest, viticulture, vineyard management, ripening heterogeneity

## Abstract

Background: Berry quality potential from a single vineyard is mainly defined based on some physicochemical parameters and subjective assessments. In this way, berry maturity variability would be a key factor affecting berry quality. Methods: This trial aimed to study the effects of the maturity variability of berries harvested from plots of low (~37,080 kg ha^−1^), middle (~12,545 kg ha^−1^), and high (~1476 kg ha^−1^) quality potential on berry and wine physicochemical parameters of Cabernet Sauvignon in two consecutive seasons. The quality potential of the plots was defined by the winemakers considering mostly yield per hectare and the final price of their wines. Results: The berry heterogeneous maturity of soluble solids and berry weight in Cabernet Sauvignon was confirmed. The coefficient of variability (CV) of berry weight of high-quality plots was high at véraison and decreased as ripening progressed, reaching CV of 19.9% at harvest. Low-quality plots showed the lowest CV of berry weight in all the studied dates, whereas high-quality plots presented the lowest CV in soluble solids content of berries, reaching a 5.1% of variability at harvest. The physicochemical parameters showed that high-quality plots were characterized by high levels of soluble solids and phenolic maturity parameters, whereas samples from low-quality plots reached high berry weight and malic acid content. Berry differences among the physicochemical parameters determined wine quality, which allowed for plots to be classified by their potential quality at harvest. Conclusions: Studying maturity variability of soluble solids and berry weight will allow for sampling to be sectorized within a vineyard to reduce the extremes of maturity that would affect wine quality and productive goals of winemakers.

## 1. Introduction

There is a strong relationship between the sale price and the quality parameters of grapes in most of the wine-producing areas in the world [1]. In the wine industry, a degree of grape maturity is a particular goal for winemakers since it allows them to produce high-quality products [2]. Regardless of the definition of grape quality, there is a continuous search for viticultural techniques to improve the wine quality, mostly in the Cabernet Sauvignon wines produced in Chile [3,4], the most important national grapevine variety.

Berry composition at harvest strongly conditions wine quality [5,6]. Harvest date is defined by a specific degree of berry maturity, which is characterized by an asynchronous development between berries within a bunch or a vine [7]. Topo-edaphoclimatic conditions and vine physiological status at flowering usually alter ovule fecundation and the resulting seed number per berry [5,6]. This physiological phenomenon is normally defined as the origin of the asynchrony of berry development [5,8]. The asynchrony of ripening is usually observed in commercial vineyards, and it has been well-described in the scientific literature [5,7,9,10].

The variability of bunch and berry ripening parameters has been usually described as a negative parameter by winemakers. In addition, this is a widespread phenomenon that is commonly accepted, and its evaluation is omitted even in several research trials published in the scientific literature [11]. The heterogeneity of berry ripening within the bunch is a factor largely documented, but there are few studies that try to understand and solve this phenomenon [11,12]. This phenomenon has been mentioned as an essential and determining factor that affects the quality and sales price of grapes and wines [12,13,14]. Berry maturity variability at harvest is a consequence of asynchronous ripening, which results in the obtention of berries with a variable chemical composition at harvest [10].

The berry ripening process within the bunch occurs at different rates and is affected by multiple factors, such as the vineyard location, bunch position in the vine, and berry location in the bunch [6]. Berry ripening asynchrony is also attributed to other factors related to vine management, such as the training system, fruit load, and pruning which alters photoassimilate production [15]. In this sense, at different berry ripening stages, it is possible to find different populations of grapes with different levels of maturity [7]. This variability is greater at the beginning of the berry ripening, and it decreases towards the harvest date [13]. A varied content of sugar can be found in the individual berries collected at harvest, which could affect the sensory and organoleptic characteristics of the produced wines [16]. Berries that are less ripe have low sugar content, contributing to high acidity and a varied extraction of phenolics from seeds and skins [17]. The wines made from heterogeneous grape populations in their technological maturity have different sensory characteristics than wines produced from berries achieving homogeneous technological maturity, even though the means of sugar concentration are similar [18]. Therefore, quantifying the maturity heterogeneity of the berries would allow winemakers to optimize wine quality, reducing or improving green harvest according to their wine-producing goals. In addition, the technological maturity heterogeneity of the berries could also affect the selling price of the grapes since this parameter is commonly used in Chile to define the harvest quality. In this fashion, it was reported in California that high-crop-price fruit numerically had lower uniformity in soluble solids and pH compared to medium- and low-crop-price fruit [12]. In this study, Cabernet Sauvignon vineyards that provide grapes with different commercial prices did not exhibit differences in soluble solids content, pH, berry weight, and anthocyanin concentration, nor more uniform fruit [12]. As of today, there is no available scientific information about this subject in Chile.

Some technicians have proposed a selective harvest to reduce this variability and improve wine quality. However, the determination of an “acceptable” level of variability of grape maturity for optimal wine quality has not yet been proposed. Therefore, the quantification of this variability would allow viticulturists and winemakers to better define the harvest date and to classify their vineyards or plots for a desired productive goal. To our knowledge, there is scarce scientific evidence confirming the relationship between the variability of berry ripening and fruit and wine quality in accordance with a classification of the potential vineyard quality that is usually performed by the wine industry at a commercial scale. Thus, the aim of this research was to study the relationship between the variability of berry maturity and the biochemical composition of grapes and wines obtained in different vineyard plots of Cabernet Sauvignon with different quality productivity potential defined by the winemakers considering their yield per hectare.

## 2. Results

### 2.1. Berry Weight and Berry-Soluble Solids Variability Throughout Ripening

The coefficient of variation (CV) expressed in (%) of the weight of the berry (g) and soluble solids (°Brix) throughout ripening in the high- (~1476 kg ha^−1^), middle- (~12,545 kg ha^−1^), and low- (~37,080 kg ha^−1^) potential quality plots are shown in Table 1. The CV of the weight of the berry ranged from 32.50 to 19.96% for high-quality plots with an average of 26.98%. The CV of the weight of the berry varied from 37.32 to 30.66% in middle-quality plots with an average of 32.90%, whereas the CV of this parameter ranged from 25.79 to 20.39% with an average of 23.77% in low-quality plots. The CV of berry-soluble solids ranged from 8.66 to 5.12% for high-quality plots with an average of 6.71%. The CV of berry-soluble solids varied from 16.00 to 7.18% in middle-quality plots with an average of 10.96%, whereas the CV of this parameter ranged from 8.60 to 8.30% with an average of 9.46% in low-quality plots. The CV of the weight and soluble solids decreased as ripening progressed (from 14 days after véraison to 1 day before harvest) from 31.87 to 23.67% and from 11.09 to 6.87%, respectively.

### 2.2. Berry Weight and Berry-Soluble Solids Evolution Throughout Ripening

**Figure 1:**
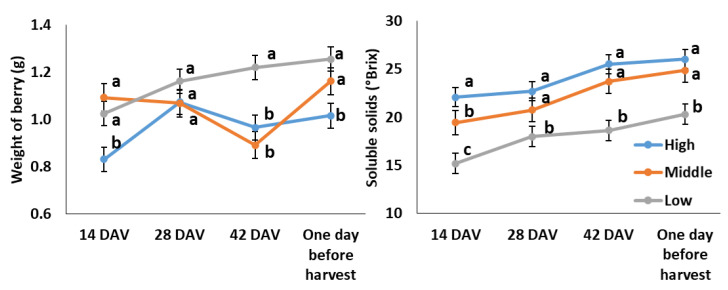
Evolution of berry weight and soluble solids in Cabernet Sauvignon samples from high-, middle-, and low-quality plots throughout ripening. Graph points corresponded to the means of the determinations obtained in each date that are shown with their standard deviation. In this, the means followed by the same lower-case letter in the same date do not differ statistically.

Berry weight (g) and berry-soluble solids (°Brix) evolution throughout ripening in high-, middle-, and low-quality potential plots are shown in Figure 1. In general terms, low-quality plots showed a higher weight of the berry in the samples of most of the studied ripening stages compared to the high-quality ones (in most of the stages, except at 28 days after véraison). The content of soluble solids in grapes increased as the ripening progressed on high-, middle-, and low-quality plots. Nevertheless, the grapes from low-quality plots showed the lowest accumulation of soluble solids throughout ripening.

### 2.3. Distribution Analysis of Weight of Berry and Berry-Soluble Solids at Harvest

**Figure 2:**
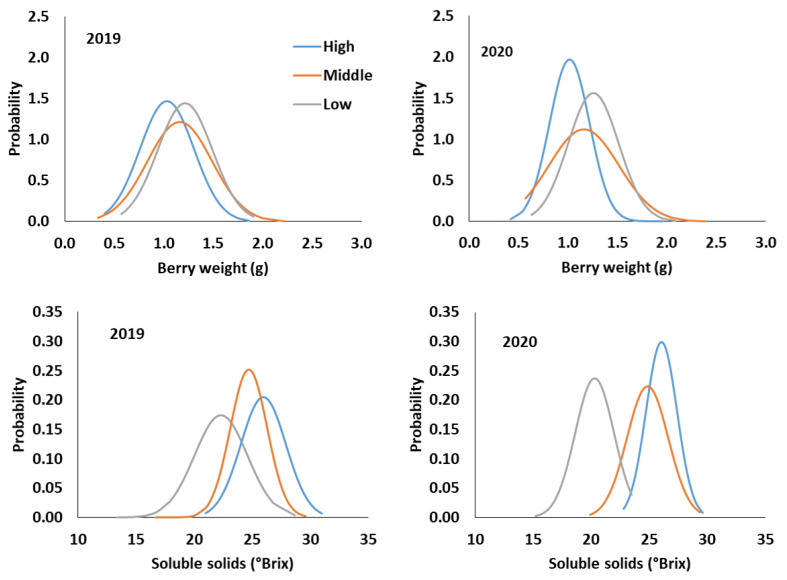
Normal distribution of berry weight (g) and berry-soluble solids (°Brix) obtained from Cabernet Sauvignon samples plots with high-, middle-, and low-quality potential in 2019 and 2020 seasons.

Berry weight (g) and berry-soluble solids (°Brix) distribution throughout ripening in high-, middle-, and low-quality potential plots in two consecutive seasons are shown in Figure 2. The highest values of berry weight corresponded to the berries obtained from low-quality plots, reaching an average value of 1.21 and 1.25 g in the 2019 and 2020 seasons, respectively, whereas the lowest berry weight was obtained from high-quality plots, reaching an average value of 1.03 and 1.02 g in 2019 and 2020 seasons, respectively.

The content of soluble solids reached the highest values in the berries obtained from high-quality plots, reaching an average value of 25.98 and 26.05 °Brix in the 2019 and 2020 seasons, respectively, whereas the lowest berry-soluble solids content was reached in berries obtained from low-quality plots, presenting an average value of 22.32 and 20.30 °Brix in the 2019 and 2020 seasons, respectively.

### 2.4. Physicochemical Parameters of Berries at Harvest

Physicochemical parameters of berries at harvest in Cabernet Sauvignon plots of high-, middle-, and low-quality potential, previously established by the commercial vineyard, are shown in Table 2. The pH and total acidity were statistically similar in the plots in the 2019 season. High-quality plots provided berries with the highest content of soluble solids, extractable anthocyanins, and skin phenols, as well as the lowest berry weight content in the 2019 season. Berries from low-quality vineyards showed the highest content of malic acid content and lower content of extractable anthocyanins and total polyphenol index (TPI) than the berries obtained from the high-quality plots in the 2019 season. The malic acid and skin phenols contents were not affected by the quality potential in the 2020 season. Low-quality plots provided berries with the lowest content of soluble solids and the highest berry weight in the 2020 season. Berries obtained from high-quality vineyards presented higher pH, extractable anthocyanins, potential anthocyanins, and TPI, as well as lower total acidity than the berries from low-quality vineyards in the 2020 season.

### 2.5. Principal Component Analysis and Correlation Matrix of Variables Analyzed in Grapes

**Figure 3:**
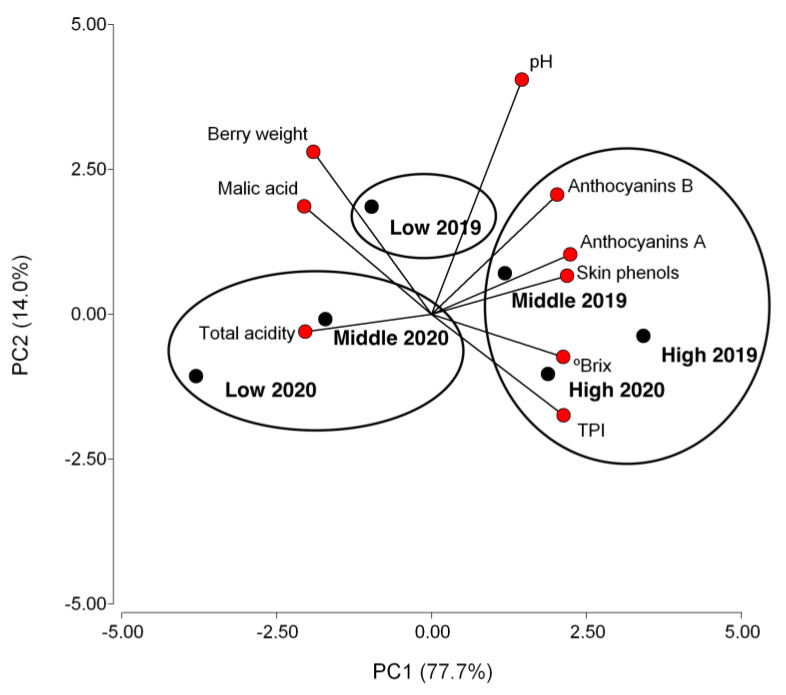
Principal component analysis was performed with the physicochemical parameters of berries obtained from Cabernet Sauvignon plots with high-, middle-, and low-quality potential in 2019 and 2020 seasons. Abbreviations: Soluble solids: °Brix. Anthocyanins A: extractable anthocyanins. Anthocyanins B: potential anthocyanins.

To classify the different samples, principal component analysis (PCA) was performed using the physicochemical parameters at harvest from berries collected from the studied Cabernet Sauvignon plots (Figure 3). PC1 and PC2 represented 77.7 and 14.0% of the total variance, respectively, representing 91.7% of all the variance. PC1 was strongly correlated (r^2^ > 0.9) to the soluble solids (+), extractable anthocyanins (+), skin phenols (+), and total polyphenol index (+), whereas none of the variables were strongly correlated to PC2. Berry data from high-quality plots in both seasons were located on the opposite side of berry data from low-quality plots, whereas the rest of the treatments were placed in the middle side on PC1.

Table 3 shows the matrix of the correlations between nine berry parameters of berries collected from Cabernet Sauvignon plots of different potential quality previously established by the commercial vineyard. Soluble solids had significant correlations with berry weight (−0.91; *p*-value < 0.05), extractable anthocyanins (0.89; *p*-value < 0.05), skins phenols (0.88; *p*-value < 0.05), and total phenolic index (0.82; *p*-value < 0.05), indicating that the berries with high soluble solids presented low berry weight and a high total phenolic index. Berry weight had significant correlations with malic acid (0.86; *p*-value < 0.05) and total phenolic index (−0.89; *p*-value < 0.05), indicating that weighted berries presented high malic acid content and low total phenolic index. Total acidity had significant correlations with malic acid (0.89; *p*-value < 0.05), extractable anthocyanins (−0.82; *p*-value < 0.05), and total polyphenol index (−0.86; *p*-value < 0.05), indicating that berries that reached high acidity presented high malic acid and low extractable anthocyanins and total polyphenol index. Malic acid had significant correlations with a total polyphenol index (−0.99; *p*-value < 0.01), indicating that berries with high malic acid content presented low total phenolic index content. Extractable anthocyanins had significant correlations with potential anthocyanins (0.95; *p*-value < 0.01), skin phenols (0.98; *p*-value < 0.01), and total polyphenol index (0.82; *p*-value < 0.05), whereas potential anthocyanins were correlated to skin phenols (0.93; *p*-value < 0.01). Moreover, skin phenols were correlated to the total polyphenol index (0.82; *p*-value < 0.05), indicating a strong relationship among the phenolic maturity in Cabernet Sauvignon berries.

### 2.6. Physicochemical Parameters of Wines

Physicochemical parameters of wines produced from the grapes obtained in Cabernet Sauvignon plots of high-, middle-, and low-quality potential, previously established by the commercial vineyard shown in Table 3. The wines elaborated from berries collected from high-quality plots reached the highest alcohol degree and total acidity, whereas the wines vinified using berries from low-quality plots had the highest pH and the lowest content of total anthocyanins and color intensity in the 2019 season. The wines elaborated from berries harvested from high-quality plots presented the highest alcohol degree and color intensity, as well as the lowest total acidity in the 2020 season. The wines vinified using berries from low-quality vineyards showed the lowest pH and total anthocyanins in the 2020 season.

## 3. Discussion

The results exposed in this trial confirmed the heterogenous character of berry maturation as was widely exposed previously by different authors [7,12,13]. The coefficient of variation (CV) indicated that from 14 days after véraison (DAV), berry weight and berry-soluble solids content tended to decrease towards harvest in the three studied plots (Figure 1 and Table 1). Similar to this, Pagay and Cheng [13] showed a high variability in berry diameter at véraison in Cabernet Franc and Concord. Then, it was reduced towards harvest. Previously, Coombe [19] and May [20] reported differences in maturity of up to 23 days between two bunches in the same vine. These differences could be related to the fruit set dynamic that affects the onset of ripening [7].

Based on the exposed results in this trial, the lowest values of variability in the soluble solids content were found in the berries obtained from high-quality plots during the sampling dates. García de Cortazar–Atauri et al. [7] reported different ripening classes in Garnacha and Syrah berries according to their soluble solids content. These authors reported variations between 9.0 and 11.5 ° Brix within a berry population, which confirms a heterogeneous maturity in terms of sugar accumulation in grapes. Gray [11] mentioned that uniform vines reached a variability of soluble solids content close to 5%, similar to the results obtained in this trial in high-quality plots. Zoecklein et al. [21] indicated that the berry-soluble solids content could be considered uniform at a percentage of variability lower than 10%. These authors suggested that the heterogeneity could increase according to the heterogeneity in the vine material used at planting that could provide different maturity of the berries in the clusters. Based on the scientific literature, the variety factor could considerably affect the percentage of variability of the soluble solids content of berries. Pagay and Cheng [13] reported a high degree of variability (14%) at harvest in the content of soluble solids of berries harvested from the Concord vines. Bishop et al. [22] reported variability of the content of soluble solids among berries in 31, 22, and 34%, among bunches in 28, 69, and 18%, and among vines in 41, 9, and 48% in Vidal Blanc, Seyval Blanc, and Pinot Gris, respectively. Our results also showed important variations in berry-soluble solids content ranging from 6.5 to 18.3 °Brix, and higher levels of variability were found in the berries from middle- and low-quality plots. The variability in the soluble solids content could be related to the intrinsic levels of auxins in the berries. In this way, Zhang et al. [23] reported high concentrations of auxins in berries at early ripening stages that decreased towards harvest. Kühn et al. [24] showed that auxin levels in berries decreased after véraison in concordance with an increase in glucose and fructose content. Since each berry develops individually, different levels of soluble solids are reached in a bunch. Therefore, high-quality plots (i.e., vineyards with a low yield) would provide conditions that result in a balanced hormonal process in berries, allowing for a more uniform maturity at harvest.

Low-quality plots reported the highest berry weight in this research trial. In general, the goal of winemakers in the wine industry for plots with high yields, regardless of berry composition, is to produce bulk wines. In contrast, high-quality plots provided berries with low weight and high phenolic content in this research trial. Rolle et al. [25] reported that small berries have a high concentration of phenolic compounds in the skin, which would be a qualitative indicator. Middle-quality plots reached the highest berry weight range in both seasons, but the range differences between low- and high-quality vineyards were similar (Figure 2). In this sense, the normal distribution of berry weight could not be suggested as a qualitative parameter of the vineyard. High-quality plots were characterized by a low range of berry-soluble solids distribution (Figure 2). These effects were probably due to the fact that high-quality plots have a better leaf-to-fruit ratio (Table 4), providing a balanced and uniform translocation of photoassimilates to berries and allowing for a homogeneous maturity at harvest in terms of soluble solids. Zoecklein et al. [21] reported a wide distribution in the content of soluble solids in berries that reached an average bellow 18 °Brix. These authors found that this difference resulted in a variation in the aroma, flavour, and phenolic compounds of the must obtained after crushing grapes. These results are similar to those exposed in this trial since low-quality plots provided a wide range of berry-soluble solids content.

The berries from high-quality plots reached higher levels of soluble solids, anthocyanin concentration, and total polyphenol index than the berries from the other plots in this research trial. These results did not agree with those reported by Calderón–Orellana et al. [12], who found no differences in soluble solids, pH, and anthocyanin concentration in berries among vineyards of different grape sell prices, a parameter that is usually used as an indicator of the potential level of quality of the vineyards. Based on our results, the high-quality plots in which the winemakers value with a higher price for their fruit were characterized by high levels of soluble solids, total polyphenol index, skin phenols, and extractable and potential anthocyanins, which are indicators of berry quality. In contrast, low-quality plots were characterized by high levels of total acidity, malic acid, and berry weight. Based on the results previously exposed, the commercial classification used in this study determined the differences in wine parameters. Contrary to this, Pineau et al. [18] reported no statistical differences among Pinot Noir wines elaborated from berries with different levels of maturity. Cuadros–Inoztroza et al. [26] reported significant differences among the wines produced with bunches with different classes of maturity. The matrix of correlations exposed in the Section 2 showed that berry-soluble solids content was correlated to berry weight, extractable anthocyanins, skin phenols, and total phenolic index, whereas berry weight was correlated to malic acid and total polyphenol index. Based on this, phenolic maturity and organic acids in berries could reach a high variability among the bunch, and more studies deserve to be performed to understand their impacts on wine quality.

Based on the results exposed in this trial, wines made from berries harvested from vineyards with high-soluble solids variability resulted in wines with lower alcohol content, anthocyanin concentration, and color intensity. In this fashion, a high proportion of unripe berries could be obtained from a population of samples with high soluble solids variability. Kontoudakis et al. [27] reported that unripe grapes decreased ethanol content, pH, anthocyanin concentration, color intensity, total phenolic index, and proanthocyanidin concentration and increased titratable acidity of Cabernet Sauvignon wines. Canals et al. [17] reported that the extraction of anthocyanins from the skins and proanthocyanidins from the skins and seeds was significantly higher when the berries reached ripeness. García de Cortázar–Atauri et al. [7] reported that high-soluble solids variability in berries did not affect wine quality. Parr et al. [28] mentioned that, regardless of the style of wine that the winemaker wants to produce, knowing the variability of ripeness could help identify possible aromas and flavors coming from the grape and to differentiate harvest for a distinct goal.

As of today, it is assumed that the homogeneity of berry maturity is more desirable than the heterogeneity of the technological parameters of grapes, but an acceptable level of heterogeneity for optimal wine production is still unknown [29]. Based on the results exposed in this trial, in Cabernet Sauvignon vineyards, the coefficient of variation of berry-soluble solids content and berry weight should reach levels close to 5 and 20%, respectively, for optimum wine production. At a technical level, the variability of berry maturity is high even in small vineyard blocks. As a consequence, within vines and vine rows, the variability of berry technological maturity is significantly (>5% at harvest date) independent of the potential quality of each vineyard or plot, as was exposed in this trial. Regarding the winemaking point of view, achieving a berry maturity homogeneity in the vineyard blocks is crucial for the production of premium or ultra-premium wines. To limit this variability, it is possible to form sub-blocks within vineyard blocks and, thus, ensure less variability in the technological maturity of the berries. In addition, quantifying the variability of the berry’s technological maturity would enable specialists to select uniform vineyard plots for winemaking, ensuring homogeneous ripeness when producing high-quality wines. Berry monitoring of the technological maturity can begin from veraison onwards, as it represents a straightforward and cost-efficient method that can be readily implemented in vineyard management practices. Most of the wineries are equipped with instruments capable of performing routine analyses, such as the one proposed in this trial. Consequently, selecting uniform sectors within the vineyard can significantly enhance wine quality, particularly in vineyards aimed to produce ultra-premium wines. This approach can also facilitate the identification of distinct vineyard plots that could contribute to the final wine blend, according to the winemaker’s specific production objectives.

## 4. Materials and Methods

### 4.1. Study Sites, Plant Material, and Experimental Design

A research trial was performed during two consecutive seasons (2019 and 2020) in six Cabernet Sauvignon plots located in different locations of three wine-growing Chilean valleys: (i) Maipo Valley, located in the Metropolitan Region; (ii) Colchagua Valley, located in the O’Higgins Region; and (iii) Curicó Valley, located in the Maule Region (Table 4). The Cabernet Sauvignon plots belong to national wine companies, and each of the six plots received differentiated classifications of potential quality according to the commercial preferences of the company defined by the winemakers (Table 1). The definition of commercial classifications of each plot was performed by the specialists and integrated mostly yield per hectare and the price of grapes, including the production system, agronomic management, the production area, and oenological potential, among others (Table 1). The classification was defined as follows: (i) low-quality plots (~37,080 kg ha^−1^); (ii) middle-quality plots (~12,545 kg ha^−1^), and high-quality plots (~1476 kg ha^−1^). In this study, the viticultural managements exposed in Table 1 were replicated during the two consecutive seasons.

The experiment was established in a completely randomized design, accounting for three treatments that corresponded to high-, medium-, and low-quality plots with two replications that consisted of two plots for each quality level, as is exposed in Table 1. The plots were randomly selected from commercial vineyards established in different Chilean valleys as is exposed in Table 1. In detail, two plots of similar yield (~1476 kg ha^−1^) were selected as replications for high-quality potential treatment in two different commercial vineyards located in the Maipo Valley, accounting for a total of four replications by treatment. Similar to this, two plots of similar yield (~12,545 kg ha^−1^) were selected as replications for the middle-quality potential treatment in two different commercial vineyards located in the Colchagua and Maipo Valleys, accounting for a total of four replications by treatment. Moreover, two plots of similar yield (~37,080 kg ha^−1^) were selected as replications for the low-quality potential treatment in two different vineyards located in the Curicó and Maipo Valleys, accounting for a total of four replications by treatment. In each plot or replication, sampling and measurements were conducted and duplicated in approximately 25 vines located in two rows of the middle zone of each of the selected plots.

### 4.2. Berry Harvest and Winemaking

Harvest was conducted when grapes reached their optimal sensorial maturity, according to the winemaker’s decision in charge of each plot. The leaves of one vine per treatment were manually extracted after the harvest, and, subsequently, they were introduced into a leaf-area meter (LI-COR, LI-3100 C, Lincoln, NE, USA) with the aim of calculating leaf-to-fruit ratio (Table 4). After harvest, the collected grapes were stored for 1 day in a cold chamber at 6 °C before processing. Then, they were destemmed and crushed to obtain the must. The winemaking process was performed according to the protocol defined by the Vine and Wine Technological Center (Universidad de Talca, Talca, Chile) that was exposed by Gutiérrez–Gamboa et al. [30].

Briefly, each replicate was introduced into 50 L tanks, so 12 tanks were filled (two tanks for each replication). Tanks were stored at 6 °C for pre-fermentative maceration for 2 days. Subsequently, the must was inoculated with a commercial yeast *Saccharomyces cerevisiae* strain BO 213 (Laffort, Bordeaux, France) to conduct the alcoholic fermentation, which occurred at 26 ± 1 °C. The alcoholic fermentation was considered finished when the must reached 2.5 g L^−1^ of residual sugar. Then, after 16 days of maceration–fermentation, the skins and seeds were manually removed, and the tanks were carried to a cold chamber at 6 °C to eliminate lees. Then, the wine samples were subjected to the analysis of oenological parameters.

### 4.3. Determinations of Physicochemical Parameters of Berries Throughout Ripening

**Figure 4:**
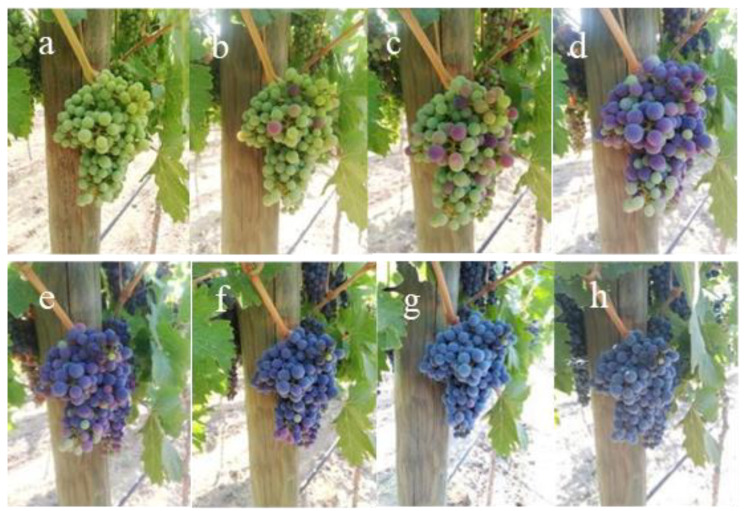
Berry maturity evolution in the same bunch at 47 (**a**), 52 (**b**), 54 (**c**), 59 (**d**), 61 (**e**), 67 (**f**), 74 (**g**), and 79 (**h**) days after fruit set in Itahue, Curicó, Curicó Valley.

The content of soluble solids and weight of berries were determined at 14, 25, and 42 days after véraison, including 1 day before harvest (Figure 4). Berry sampling was performed, obtaining four replicates of 50 berries by repetition and treatment (200 berries per repetition) at each of the studied ripening stages. In that sense, a total of 25 berries were obtained from 8 different bunches that were randomly selected in each of the determined replications. The physicochemical parameters were determined in the individual berries. The content of soluble solids (°Brix) was determined using a digital refractometer (ATAGO, Saitama, Japan), whereas weight of the berries was analyzed using an analytical balance (Cubis Precision Balance, Sartorius, Göttingen, Germany).

### 4.4. Determinations of Physicochemical Parameters of Berries at Harvest

At harvest, approximately 20 kg of berries were obtained from each plot and were stored for 1 day in a cold chamber at 6 °C before processing. Subsequently, weight of berries was determined in 100 berries randomly collected from the 20 kg harvested using an analytical balance (Cubis Precision Balance, Sartorius, Göttingen, Germany). The physicochemical parameters, such as soluble solids, pH, total acidity (g L^−1^ of sulfuric acid), and malic acid were determined according to the International Organization of Vine and Wine (OIV) methodologies [31] in a must sample obtained from the crushing of 500 g of berries. The determinations of phenolic maturity, such as the potential and extractable anthocyanins (at pH 1.0 and pH 3.2, respectively), skin phenol content, and total polyphenol index (TPI) were determined according to the methodologies proposed by Glories and Augustin [32]. Briefly, skin phenol content was determined by spectrophotometry at an absorbance of 520 nm (in a solution at pH 3.2), whereas TPI was determined by spectrophotometry at an absorbance of 280 nm.

### 4.5. Determinations of Physicochemical Parameters of Wines

The physicochemical determinations in the wines were performed immediately after wine bottling in both studied seasons. Thus, alcohol degree, pH, total acidity (g L^−1^ sulfuric acid), volatile acidity (g L^−1^ acetic acid), total and free sulfur dioxide, and reducing sugars were analyzed according to OIV [31] methodologies in both studied seasons. Total anthocyanins and color intensity were determined in wines by spectrophotometry according to the methods exposed by Bordeu and Scarpa [33].

### 4.6. Statistical Analysis

The coefficient of variation (CV) was used to define the variability of each measured parameter in the established treatments. The CV was calculated in percentage (%) as the ratio of the standard deviation to the mean. The statistical analysis of physicochemical parameters and the rest of the determinations in grapes and wines was performed using a variance analysis (one-way ANOVA) by Statgraphics Centurion XVI.I (The Plains, VA, USA). The differences among samples were compared using the LSD test at 95% probability level. The means of each determined variable was the result of four different data. Principal component analysis (PCA) was performed using all data to classify treatments and parameters. PCA was performed using the Infostat 2014 software.

## 5. Conclusions

The coefficient of variation of the berry-soluble solids content and berry weight changed according to the potential quality of the plot defined by the winemakers. Based on this, the maturity variability of the berries could be a potential qualitative indicator of the vineyard. Therefore, it is possible to sectorize berry sampling within a vineyard to reduce the extremes of maturity in terms of soluble solids and berry weight and optimize wine quality and productive goals of the winemakers.

## Figures and Tables

**Table 1:** Coefficient of variation (%) of the weight of berry (g) and soluble solids (°Brix) in Cabernet Sauvignon plots of high-, middle-, and low-quality potential throughout ripening.

Quality Potential	Weight of Berry				Soluble Solids (°Brix)			
	14 DAV	28 DAV	42 DAV	One Day before Harvest	14 DAV	28 DAV	42 DAV	One Day before Harvest
High	32.51%	27.15%	28.29%	19.96%	8.66%	7.75%	5.31%	5.12%
Middle	37.32%	29.39%	30.21%	30.66%	16.00%	11.62%	9.04%	7.18%
Low	25.79%	26.83%	22.05%	20.39%	8.60%	10.14%	10.80%	8.30%

Abbreviations: DAV: days after véraison. Mean of each variable and ripening stage correspond to an average of 400 individual berries (n = 400).

**Table 2:** Physicochemical parameters of berries at harvest in Cabernet Sauvignon vineyards of different potential quality previously established by the commercial vineyard.

Quality Potential	Soluble Solids (°brix)	Berry Weight (g)	pH	Total Acidity (g L^−1^)	Malic Acid (g L^−1^)	Extractable Anthocyanins (g L^−1^)	Potential Anthocyanins (g L^−1^)	Skins Phenols (mg kg^−1^)	TPI
Season 2019									
High	26.78 c	0.92 a	3.67	4.18	1.24 a	591 b	860 b	14.36 b	50.69 b
Middle	24.53 b	1.13 b	3.76	3.93	1.39 a	524 a	704 a	11.85 a	44.25 ab
Low	21.73 a	1.33 c	3.8	4.62	1.65 b	480 a	750 ab	11.10 a	39.38 a
Significance	***	***	ns	ns	**	*	*	**	*
Season 2020									
High	24.43 b	1.01 a	3.61 b	3.80 a	1.13	508 b	709 b	11.62	51.01 b
Middle	23.56 b	1.38 b	3.55 a	5.04 b	1.66	433 ab	628 a	9.91	38.34 a
Low	20.59 a	1.25 c	3.33 a	5.25 b	1.69	369 a	465 a	8.84	37.71 a
Significance	**	***	*	**	ns	*	**	ns	**

TPI: Total phenolic index. Total acidity was expressed as sulfuric acid. *** (*p*-value: ≤0.001), ** (*p*-value: ≤0.01) and * (*p*-value: ≤0.05). Mean of each variable corresponds to an average of four samples (n = 4). Means followed by the same lower-case letter in the same column do not differ by the LSD test at the 5% level of significance.

**Table 3:** Matrix of the correlations between berry physicochemical parameters of berries at harvest in Cabernet Sauvignon vineyards of different potential quality previously established by the commercial vineyard (above the diagonal).

	Soluble Solids	Berry Weight	pH	Total Acidity	Malic Acid	EA	PA	Skin Phenols	Total Phenolic Index
Soluble solids	1								
Berry weight	−0.91 *	1							
pH	0.48	−0.13	1						
Total acidity	−0.70	0.63	−0.68	1					
Malic acid	−0.78	0.86 *	−0.35	0.89 *	1				
EA	0.89 *	−0.72	0.74	−0.82 *	−0.77	1			
PA	0.78	−0.56	0.79	−0.69	−0.61	0.95 **	1		
Skin phenols	0.88 *	−0.75	0.64	−0.74	−0.75	0.98 **	0.93 **	1	
Total phenolic index	0.82 *	−0.89 *	0.35	−0.86 *	−0.99 **	0.82 *	0.68	0.82 *	1

Abbreviations: EA: extractable anthocyanins. PA: potential anthocyanins. Data in bold correspond to a significant relationship. In the table, ** and * indicate significance at 1 and 5% of probability, respectively, according to *t*-test.

**Table 4:** Location of Cabernet Sauvignon vineyards of different potential quality previously established by the commercial vineyard.

Quality Potential	Plot	Valley	Trellis System	Pruning System	Canopy Management	Crop Management	Average Pruning Weight (kg m^−1^)	Leaf to Fruit Ratio	Average Yield (kg ha^−1^)
High	1	Maipo	VSP *	Spur	Bud rubbing, tucking, and wire lifting	One per season	0.36	1.31	1343
	2	Maipo	VSP	Spur	Bud rubbing, tucking, and wire lifting	One per season	0.42	1.27	1608
Middle	3	Colchagua	VSP	Spur	Bud rubbing, tucking, and wire lifting	None	0.79	1.21	10,500
	4	Maipo	VSP	Spur	Bud rubbing, tucking, and wire lifting	One per season	1.5	1.19	14,590
Low	5	Maipo	Pergola	Cane	Bud rubbing, tucking, and wire lifting	None	1.49	1.13	38,580
	6	Curicó	Double VSP	Spur	Trimming	None	1.67	1.17	35,580

* VSP: vertical shoot position system. Pruning weight and yield were exposed as the average of the two seasons.

## Data Availability

The data presented in this study are available on request from the corresponding and the first author.
